# Food decisions of an omnivorous thrips are independent from the indirect effects of jasmonate-inducible plant defences on prey quality

**DOI:** 10.1038/s41598-018-38463-w

**Published:** 2019-02-11

**Authors:** Livia M. S. Ataide, Cleide R. Dias, Bernardus C. J. Schimmel, Thijs van Erp, Angelo Pallini, Merijn R. Kant

**Affiliations:** 1 Department of Evolutionary and Population Biology, Institute for Biodiversity and Ecosystem Dynamics, University of Amsterdam, Science Park 904, 1098 XH Amsterdam, The Netherlands; 2 Department of Entomology, Federal University of Viçosa, Peter Henry Rolfs s/n, 36570-000 Viçosa, Minas Gerais Brazil

## Abstract

Plant defensive substances can affect the quality of herbivores as prey for predators either directly or indirectly. Directly when the prey has become toxic since it ingested toxic plant material and indirectly when these defences have affected the size and/or nutritional value (both quality parameters) of prey or their abundance. To disentangle direct and indirect effects of JA-defences on prey quality for predators, we used larvae of the omnivorous thrips *Frankliniella occidentalis* because these are not directly affected by the jasmonate-(JA)-regulated defences of tomato. We offered these thrips larvae the eggs of spider mites (*Tetranychus urticae* or *T*. *evansi*) that had been feeding from either normal tomato plants, JA-impaired plants, or plants treated with JA to artificially boost defences and assessed their performance. Thrips development and survival was reduced on the diet of *T*. *evansi* eggs relative to the diet of *T*. *urticae* eggs yet these effects were independent from the absence/presence of JA-defences. This indicates that the detrimental effects of tomato JA-defences on herbivores not necessarily also affects their quality as prey.

## Introduction

Plants have evolved a multitude of defence traits to resist being consumed. Some of these defences are constitutive, i.e. traits displayed irrespective of the presence of herbivores or pathogens, while others are induced, i.e. traits displayed specifically upon attack^1,2^. Plant responses to herbivory have been studied in detail and roughly two types of direct defences can be distinguished: (1) physical defences that hamper herbivore behaviour (e.g. foraging or oviposition) and (2) chemical defences that affect the herbivore’s physiology, for example via toxins that act on their nervous system or enzymes that inhibit food digestion in the gut, and therefore slow down development and population growth. Besides direct defences, plants can also make use of indirect defences, which are established by attracting and arresting natural enemies of herbivores^1–3^. Defences against herbivores are primarily regulated by the plant hormone jasmonic acid (JA) and its derivatives, in particular the main biologically active conjugate jasmonic acid - isoleucine (JA-Ile)^4,5^. The effect of the plant’s JA defences on herbivores can be observed at different levels of the interaction and, for example, decrease the amount of feeding damage by the herbivore^6^, decrease its reproduction^7,8^, slow down its development^9^, decrease its survival^10,11^ and suppress its population growth^12^. Whether defences actually enhance a plant’s resistance is determined by the attacker, because it may have evolved counter-adaptations, such as the ability to conjugate, degrade, secrete and/or sequester plant toxins^13–15^.

Plant defences were found not only to negatively affect herbivores but also the natural enemies that consume them^16–19^. Often such effects were found to act directly, e.g. leaf hairs that physically hinder leaf dwelling herbivores also can hinder leaf dwelling carnivores^20^ or toxins that affect the herbivores ingesting these also intoxicate the parasitoids of these herbivores^16^. Other effects are indirect and occur because plant defences affect prey abundance^21^, prey nutritional value or size^22,23^. For instance, the induction of JA defences influences the growth rate of herbivores from different feeding guilds negatively such as caterpillars^7,12^, aphids^11,12,24,25^, spider mites^8,26^ and thrips^12,27^. Consequently, this influences the amount of prey, or prey items^8^, available for predators to eat, thereby lowering performance of the latter indirectly^28^. Importantly, induction of JA defences lowered not only population size, but also the size of the herbivore itself^9^ and the size of their eggs and other juvenile stages^29^. Lastly, JA mediates changes in resource allocation thereby lowering the nutritional quality of the plant^30–32^. Herbivores performance is affected by the quality of the host plant^33^ as well as the performance of predators is affected by the quality of the prey^22,23,34^. As a rule of thumb, the direct effects are those that carnivores in principle can adapt to (such as phytotoxins or structural barriers) while the indirect effects are those to which carnivores cannot adapt like prey abundance, nutritional value or size.

Like with carnivorous predators, plant defences can also directly and indirectly affect the performance and behaviour of zoophytophagous omnivores^35,36^. Even more so, because omnivores may feed from the plant as well, they are exposed to the sum of defences induced by themselves and by their prey^37–40^. However, due to their life style, there may be more opportunities for omnivores than for herbivores or carnivores to avoid plant defences. Indeed, omnivores are known to switch diet depending on its nutritional value in order to balance their food intake and, hence, maximize their fitness^36,41–43^. For example, the western flower thrips, *Frankliniella occidentalis*, dramatically reduces feeding from leaf material while increasing the consumption of animal prey when plant defences are induced^29^.

Direct and indirect effects of plant defences are experimentally difficult to disentangle^44,45^ because it requires a carnivore or omnivore that can cope with the plant’s defences in order to estimate the impact of the indirect effects. To assess the indirect effects of plant defences on prey quality we made use of the omnivore *Frankliniella occidentalis*. This species is a worldwide pest on ornamental plants and crops as it has a short generation time, high fecundity, great dispersal potential, and readily feeds from leaves, flowers and pollen of multiple plant species as well as on egg-, juvenile- and adult stages of various predators^46,47^ and herbivores, including spider mites^48^. When *F*. *occidentalis* feeds on tomato or Arabidopsis it induces JA-defences^6,12,49–51^. Interestingly, while adult *F*. *occidentalis* appeared to be sensitive to these naturally induced JA-defences^49,51^, their larvae appeared to tolerate these defences - unless these were artificially boosted^6,52^.

**Figure 1:**
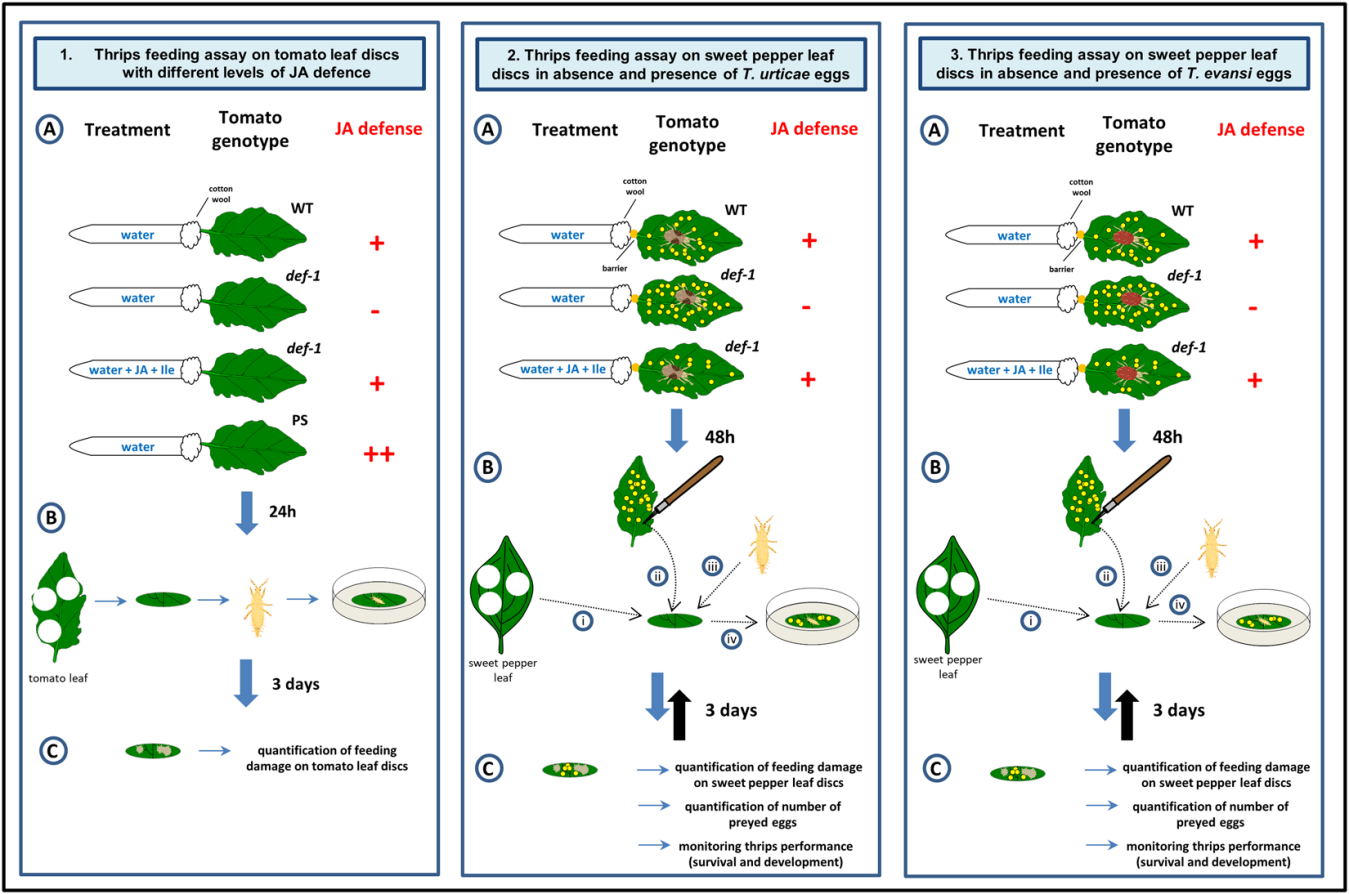
Schematic overview of the experiments carried out to assess the feeding behaviour of larvae of *Frankliniella occidentalis* when these were offered low-quality plant tissue in absence and presence of alternative food (spider mite eggs or pollen). (1) Quantification of thrips feeding damage on leaflets of tomato genotypes with varying levels of JA defences. (1 A) Detached tomato leaflets were placed with their petiole in tap water or tap water + JA + Ile. (1B) After 24 h, two or three leaf discs (d = 9 mm) were prepared from each leaflet and individually placed on wet cotton wool in a petri dish and a single thrips larva was introduced to each leaf disc. (1C) After three days, the larvae were removed and the leaf disc was scanned, to quantify the amount of thrips feeding damage afterwards. (2) and (3) Assessment of thrips feeding behaviour and performance on sweet pepper leaves provided with eggs of spider mites produced on tomato genotypes with varying levels of JA defences. Detached tomato leaflets were placed with their petiole in tap water or tap water + JA + Ile and were each infested with female adult *T*. *urticae* (2 A) or *T*. *evansi* (3 A). (2B) and (3B) After 48 h, sweet pepper leaf discs (d = 9 mm) were made (i) and eggs produced by spider mites on either WT tomato (‘induced eggs’), *def-1* (‘uninduced eggs’) or JA-treated *def-1* (‘boosted eggs’) were transferred onto each leaf disc (ii). A single thrips larva was introduced to each leaf disc (iii) and placed on a petri dish filled with water and cotton wool (iv). Sweet pepper leaf discs without spider mite eggs as well as discs supplemented with pollen were used as controls (not shown in the Fig). (2C) and (3C) Larval food intake (leaf area damaged, number of mite eggs eaten), developmental stage and survival were assessed for a period of 15 days. Each larva was transferred to a new, but identically treated, leaf disc every three days (back arrow).

Hence, we monitored the development and survival of thrips larvae feeding from spider mite prey obtained from plants with or without defences. To do so, we first validated the larvae to be insensitive to tomato JA-defences (Fig. 1). Subsequently, we offered them eggs from either the defence inducing spider mite *T*. *urticae*^53^ or the defence suppressing spider mite *T*. *evansi*^54^ after these had been feeding from either normal (inducible) tomatoes, from JA-biosynthesis mutant defenceless (*def-1*)^55^ or from plants in which defences were artificially boosted by treating them with JA^8^ (Fig. 1). Because we wanted to exclude direct effects of plant quality on thrips performance, we offered spider mite eggs to thrips using sweet pepper leaves since it is well established that sweet pepper is a low-quality host for thrips negatively affecting its development, survivorship, fecundity and longevity^45,47,56,57^. Under these conditions several thrips life history characteristics were monitored to assess the extent to which our thrips strain is affected by indirect effects on prey quality.

## Results

### Thrips feeding assay on detached leaflets of WT, def-1, JA-treated def-1, and PS tomato plants

**Figure 2:**
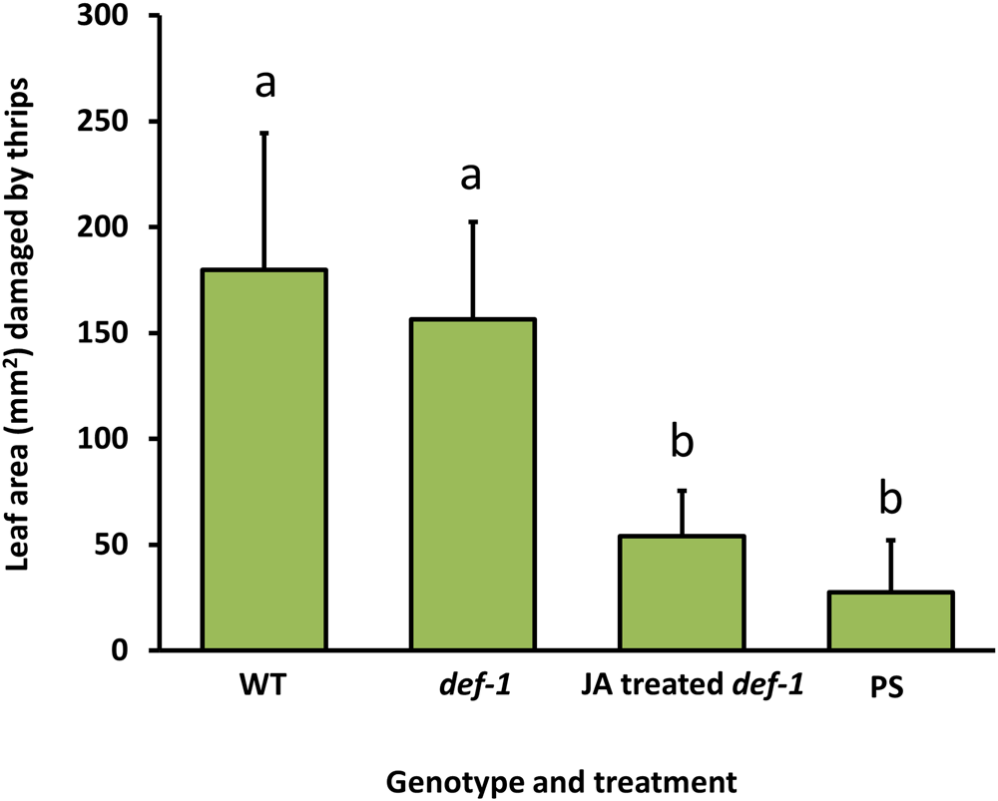
*Frankliniella occidentalis* larvae are tolerant to naturally induced jasmonic acid (JA)-regulated defences, but not to artificially boosted JA defences. The figure shows the average (+SEM) leaf area damaged by individual thrips larvae after feeding for three days on leaf tissue of different tomato genotypes, i.e. wild type (WT), a JA-impaired mutant (*def-1*) and transgenic *35 S::prosystemin* (PS). *def-1* is impaired in mounting JA regulated defences upon herbivory, whereas PS constitutively displays strong JA defence responses. The JA defences were artificially boosted in *def-1* by exogenous application of JA and Ile (JA-treated *def-1*). Different letters above the bars indicate significant differences at a level of *P* ≤ 0.05, after applying a linear mixed-effects model followed by contrast analyses.

To verify that larvae of our *F*. *occidentalis* strain are tolerant to JA defences, we quantified the amounts of feeding damage they had inflicted on leaf tissue of WT, *def-1*, JA-treated *def-1* and PS plants (Fig. 2). The amounts of leaf tissue damaged due to larval feeding varied significantly among the four treatments (LMER: χ2 _[3,6]_ = 7.5; *P* ≤ 0.05). Leaf tissue from WT and *def-1* plants had incurred about three to six times more damage than JA-treated *def-1* and *PS* leaf tissue had (LMER: χ2 _[1,4]_ = 6.6; *P* < 0.009). Thrips larvae caused similar amounts of damage on WT versus *def-1* (LMER: χ2 _[1,5]_ = 0.6; *P* = 0.4) as well as on JA-treated *def-1* versus PS leaf tissue (LMER: χ2 _[1,5]_ = 1.2; *P* = 0.7).

### Amount of thrips-inflicted feeding damage on sweet pepper leaf discs in absence and presence of alternative food (induced-, uninduced- or boosted spider mite eggs or pollen)

**Figure 3:**
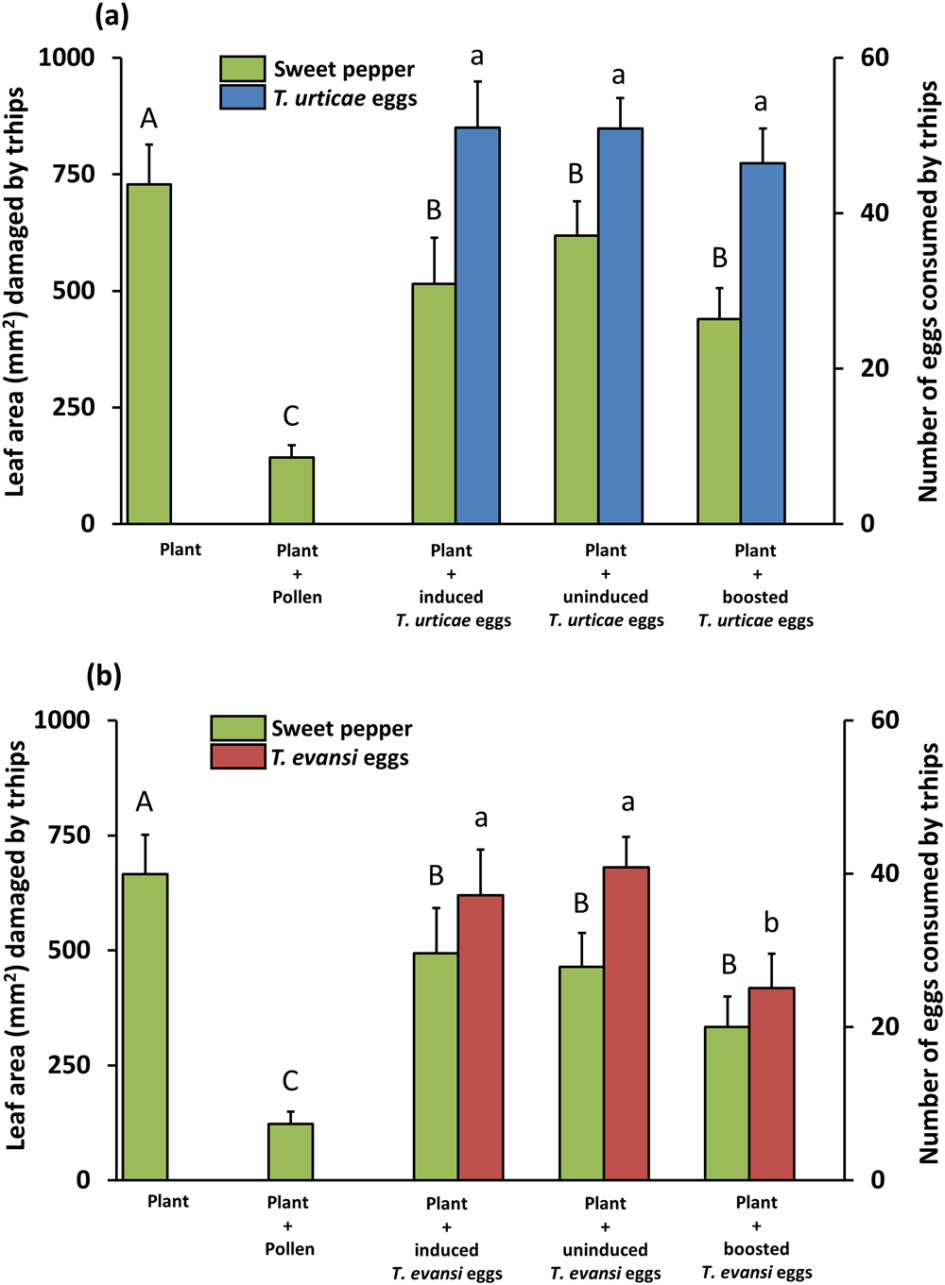
Feeding behaviour of *Frankliniella occidentalis* larvae on sweet pepper plants in absence and presence of additional food. Thrips larvae were individually placed onto sweet pepper leaf tissue with or without either broadleaf cattail pollen or spider mite eggs as additional food source. Spider mite eggs were from jasmonic acid (JA) defence-inducing *Tetranychus urticae* (**a**) or defence-suppressing *Tetranychus evansi* (**b**) and were produced on two tomato genotypes, i.e. on wild type plants (‘induced eggs’) and on *def-1* mutants impaired in mounting JA-regulated defences upon herbivory (’uninduced eggs’). Additionally, mite eggs were obtained from mites feeding on JA-treated *def-1* (‘boosted eggs’). The figure shows the average (+SEM) leaf area damaged (green bars) as well as the average (+SEM) number of spider mite eggs consumed (blue bars for *T*. *urticae* eggs, red bars for *T*. *evansi* eggs) by individual thrips larvae after feeding for 15 days on each of the diets. Different letters above the bars (capital letters for leaf damage, lowercase letters for mite egg consumption) indicate significant differences at a level of *P* ≤ 0.05, after applying a linear mixed-effects model followed by contrast analyses.

To assess the extent to which thrips larvae are affected by indirect effects of plant JA-defences - i.e. via changes in the nutritional value and/or size of their prey - we offered eggs produced by spider mites on either WT (‘induced eggs’), *def-1* (‘uninduced eggs’) or JA-treated *def-1* tomato plants (‘boosted eggs’) as prey items to our JA-defence-tolerant thrips larvae on leaf discs of sweet pepper plants, i.e. a poor-quality host plant. First, we monitored thrips feeding behaviour on sweet pepper leaf discs (Fig. 3). Thrips larvae caused most damage to sweet pepper leaf discs when no alternative food was available and only a little bit of damage (about five times less) when high quality alternative food (pollen) was present (Fig. 3, green bars; for Fig. 3a, LMER: χ2 _[1,4]_ = 57.6; *P* < 0.001; for Fig. 3b, LMER: χ2 _[1,4]_ = 51.3; *P* < 0.001). Pollen is considered as a high-quality food source given the high intrinsic rate of population increase of *F*. *occidentalis* when feeding on it^41,58^. Therefore, the treatments for which we offered sweet pepper leaf material with or without pollen served as positive and negative benchmarks, respectively, for the treatments with spider mite eggs.

When mite eggs were present as alternative food, thrips also damaged the leaf discs less than when no alternative food was present and this was independent of the source of the eggs (for Fig. 3a, LMER: χ2 _[1,4]_ = 6.0; *P* = 0.01; for Fig. 3b LMER: χ2 _[1,4]_ = 5.6; *P* = 0.02), yet the amount of damage was still three to four times more than in the presence of pollen (for Fig. 3a, LMER: χ2 _[1,4]_ = 61.8; *P* < 0.001; for Fig. 3b, LMER: χ2 _[1,4]_ = 37.4; *P* < 0.001). There was a trend towards lower damage levels in leaf discs with boosted eggs, but differences were not statistically significant (for Fig. 3a, LMER: χ2 _[1,6]_ = 1.1; *P* = 0.29; for Fig. 3b, LMER: χ2 _[1,6]_ = 2.4; *P* = 0.12).

### Thrips predation on induced-, uninduced- and boosted spider mite eggs

To assess the extent to which thrips larvae are affected by indirect effects of plant JA-defences - i.e. via changes in the nutritional value and/or size of their prey - we offered eggs produced by spider mites on either WT (‘induced eggs’), *def-1* (‘uninduced eggs’) or JA-treated *def-1* tomato plants (‘boosted eggs’) as prey items to our JA-defence-tolerant thrips larvae on leaf discs of sweet pepper plants. Here, we monitored thrips feeding behaviour on spider mite eggs (Fig. 2). Whereas thrips larvae consumed equal numbers of *T*. *urticae* eggs across the three treatments (Fig. 3a; blue bars; LMER: χ2 _[2,5]_ = 0.9; *P* = 0.64), they consumed significantly less boosted *T*. *evansi* eggs as compared to induced and uninduced eggs (Fig. 3b; red bars; LMER: χ2 _[1,4]_ = 7.4; *P* = 0.006). There was no significant interaction between predation of spider mite eggs and amount of leaf damage (for Fig. 3a, LMER: χ2 _[2,5]_ = 0.34; *P* = 0.84; for Fig. 3b: LMER: χ2 _[2,8]_ = 0.06; *P* = 0.97).

### Thrips performance on sweet pepper leaflets in absence and presence of alternative food (induced-, uninduced- or boosted spider mite eggs or pollen)

**Figure 4:**
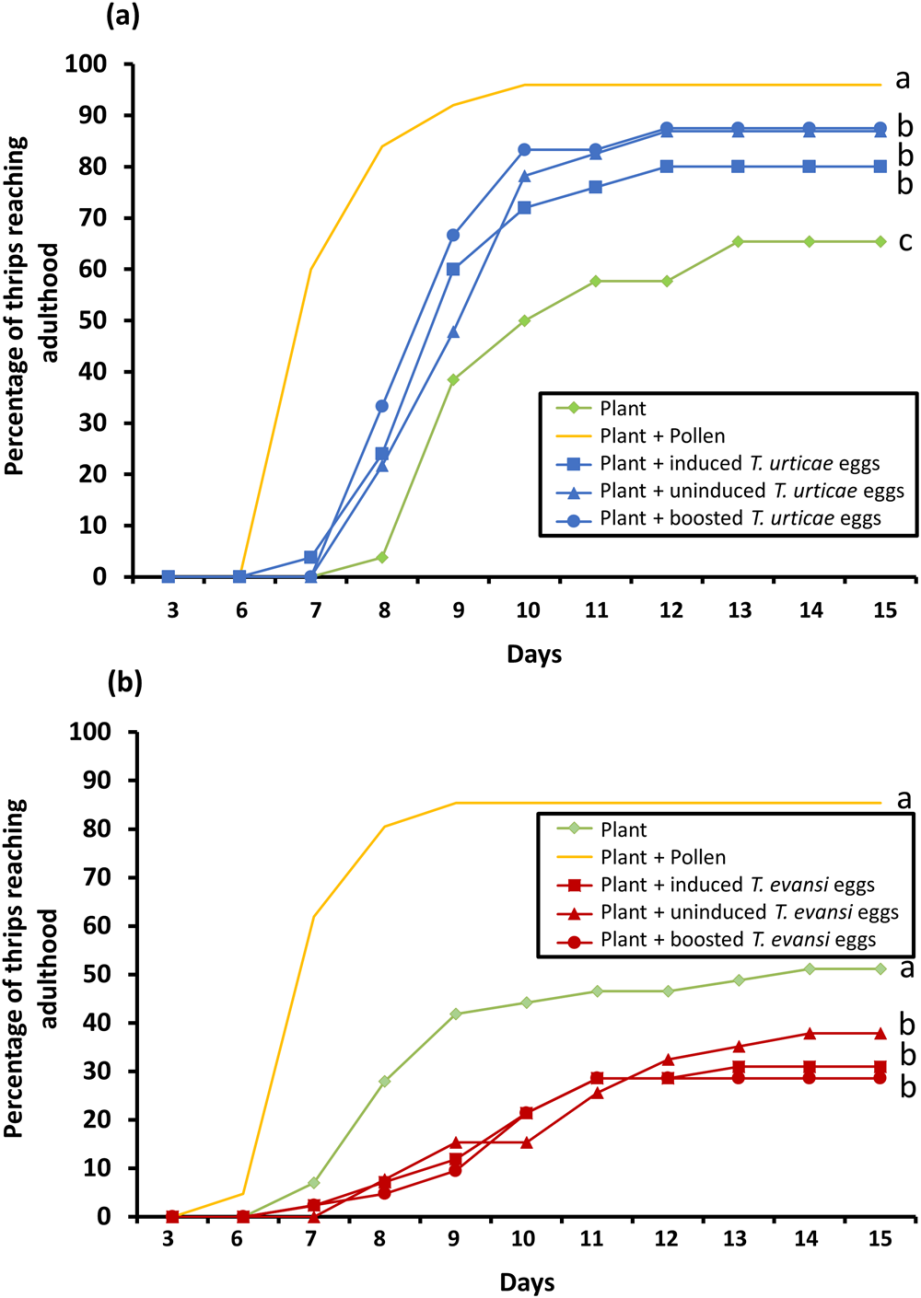
Percentage of *Frankliniella occidentalis* larvae reaching adulthood on sweet pepper plants in absence and presence of additional food. Thrips larvae were individually placed onto sweet pepper leaf tissue with or without either broadleaf cattail pollen or spider mite eggs as additional food source. Spider mite eggs were from jasmonic acid (JA) defence-inducing *Tetranychus urticae* (**a**) or defence-suppressing *Tetranychus evansi* (**b**) and were produced on two tomato genotypes, i.e. on wild type plants (‘induced eggs’) and on *def-1* mutants impaired in mounting JA-regulated defences upon herbivory (’uninduced eggs’). Additionally, mite eggs were obtained from mites feeding on JA-treated *def-1* (‘boosted eggs’). The figure shows the average percentage of L1 thrips larvae reaching adulthood as function of the time (in days) spent on each of the diets. Different letters next to the lines indicate significant differences at a level of *P* ≤ 0.05, after applying a cox mixed-effects model followed by contrast analyses.

In addition to assessing thrips feeding behaviour, we simultaneously monitored other performance parameters, such as the percentage of thrips that reached adulthood, percentage of surviving thrips and larvae-to-adult developmental time. With *T*. *urticae* eggs as additional food source, approximately 80–90% of the larvae reached adulthood within 15 days of the start of the experiment, with no significant differences among induced-, uninduced- and boosted eggs (Fig. 4a; CoxME: χ2 = 0.63; *d*.*f*. = 1; *P* = 0.43). Compared with the treatments containing *T*. *urticae* eggs, the percentage decreased to around 65% when only plant material was offered (CoxME: χ2 = 6.7; *d*.*f*. = 1; *P* = 0.009), while the percentage increased to over 90% with pollen as additional food source (CoxME: χ2 = 38.7; *d*.*f*. = 1; *P* < 0.001). By contrast, with *T*. *evansi* eggs as additional food source, only about 30–40% of the thrips larvae reached adulthood within 15 days of the start of the experiment, again with no significant differences among induced-, uninduced- and boosted eggs (Fig. 4b; CoxME: χ2 = 0.58; *d*.*f*. = 1; *P* = 0.44). When compared with the benchmark controls, the percentage reaching adulthood increased to around 50% when no additional food was available to the larvae (CoxME: χ2 = 3.9; *d*.*f*. = 1; *P* = 0.04) and to about 85% when pollen was available (CoxME: χ2 = 24.4; *d*.*f*. = 1; *P* < 0.001).

**Figure 5:**
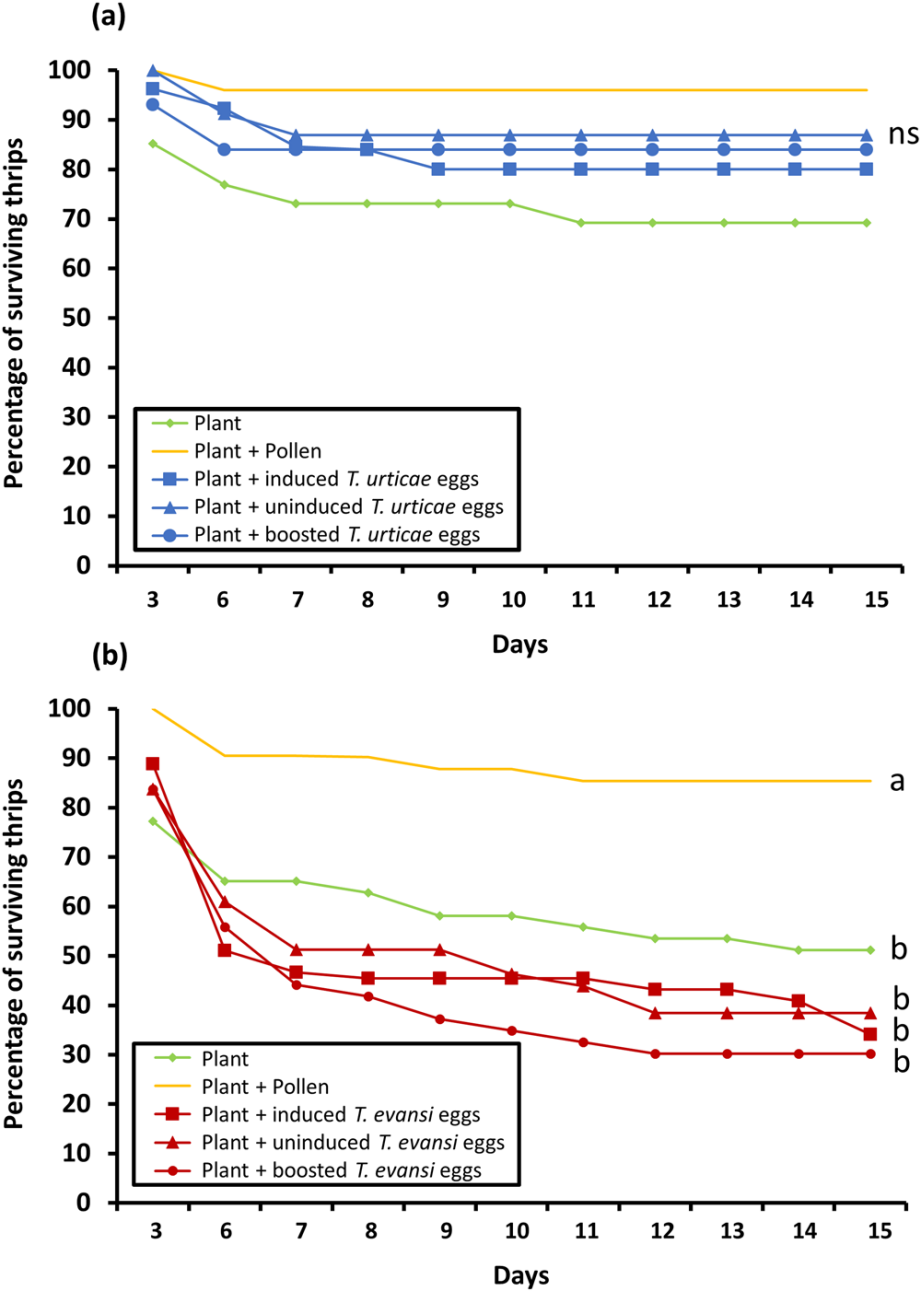
Percentage of *Frankliniella occidentalis* larvae surviving on sweet pepper plants in absence and presence of additional food. Thrips larvae were individually placed onto sweet pepper leaf tissue with or without either broadleaf cattail pollen or spider mite eggs as additional food source. Spider mite eggs were from jasmonic acid (JA) defence-inducing *Tetranychus urticae* (**a**) or defence-suppressing *Tetranychus evansi* (**b**) and were produced on two tomato genotypes, i.e. on wild type plants (‘induced eggs’) and on *def-1* mutants impaired in mounting JA-regulated defences upon herbivory (’uninduced eggs’). Additionally, mite eggs were obtained from mites feeding on JA-treated *def-1* (‘boosted eggs’). The figure shows the average percentage of surviving thrips as a function of the time (in days) spent on each of the diets. Different letters next to the lines indicate significant differences at a level of *P* ≤ 0.05, after applying a cox mixed-effects model followed by contrast analyses. ns, not significant.

Overall, thrips survival curves (Fig. 5) showed very similar patterns as those of the percentage of thrips reaching adulthood, yet for the experiments with *T*. *urticae* eggs as additional food source no statistically significant differences were found between any of the treatments (Fig. 5a; CoxME: χ2 = 1.18; *d*.*f*. = 4; *P* = 0.88). In the experiments using *T*. *evansi* eggs, thrips survival was lowest (around 30%) after feeding on a diet consisting of sweet pepper leaf with boosted eggs, but it was not statistically different from the other two treatments with eggs as additional food (Fig. 5b; CoxME: χ2 = 1.44; *d*.*f*. = 1; *P* = 0.22). Thrips survival was higher when no additional food was provided (reaching about 50%), yet it did not test statistically different from the treatments with *T*. *evansi* eggs (CoxME: χ2 = 2.63; *d*.*f*. = 1; *P* = 0.10). Significantly more thrips (approximately 90%) survived with pollen as additional food (CoxME: χ2 = 13.6; *d*.*f*. = 1; *P* < 0.001).

**Figure 6:**
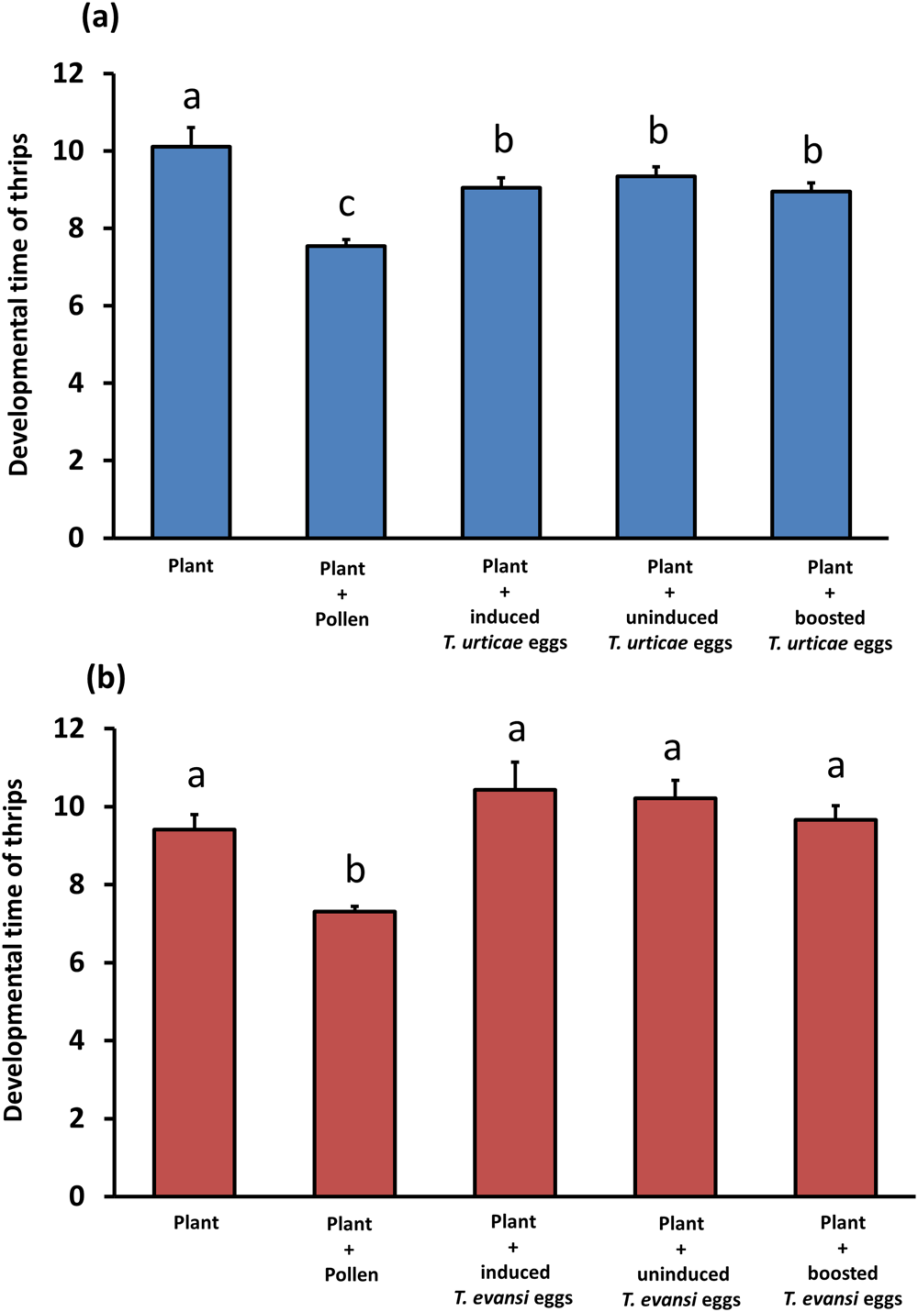
Developmental time of *Frankliniella occidentalis* on sweet pepper plants in absence and presence of additional food. Thrips larvae were individually placed onto sweet pepper leaf tissue with or without either broadleaf cattail pollen or spider mite eggs as additional food source. Spider mite eggs were from jasmonic acid (JA) defence-inducing *Tetranychus urticae* (**a**) or defence-suppressing *Tetranychus evansi* (**b**) and were produced on two tomato genotypes, i.e. on wild type plants (‘induced eggs’) and on *def-1* mutants impaired in mounting JA-regulated defences upon herbivory (’uninduced eggs’). Additionally, mite eggs were obtained from mites feeding on JA-treated *def-1* (‘boosted eggs’). The figure shows the average (+SEM) time required for L1 larvae to reach adulthood when feeding on each of the diets. Different letters above the bars indicate significant differences at a level of *P* ≤ 0.05, after applying a cox mixed-effects model followed by contrast analyses.

Taking into account only the thrips that survived until the end of the experiment (15 days), the average time required for larvae (L1) to reach adulthood was calculated. Compared with the ‘plant tissue only’ treatment, using *T*. *urticae* eggs as additional food reduced thrips’ developmental time with roughly 10% (Fig. 6a; CoxME: χ2 = 7.8; *d*.*f*. = 1; *P* = 0.005), no matter the source of the eggs (CoxME: χ2 = 0.66; *d*.*f*. = 1; *P* = 0.41). Developmental time was reduced even further (in total with over 20%) when thrips were allowed to feed from pollen instead of *T*. *urticae* eggs (CoxME: χ2 = 25.8; *d*.*f*. = 1; *P* < 0.001). Similarly, in the experiments with *T*. *evansi* eggs, thrips reached adulthood fastest when pollen was available (Fig. 6b; CoxME: χ2 = 50.3; *d*.*f*. = 1; *P* < 0.001), while no differences were detected in developmental time between the three treatments in which *T*. *evansi* eggs were the additional food source (CoxME: χ2 = 0.05; *d*.*f*. = 1; *P* = 0.81). Finally, on average thrips needed as much time to reach adulthood on sweet pepper plants without additional food as on plants supplemented with *T*. *evansi* eggs (CoxME: χ2 = 0.002; *d*.*f*. = 1; *P* = 0.96).

## Discussion

By using *F*. *occidentalis* larvae who are tolerant to tomato JA-regulated defences (Fig. 2), we showed that the quality of its animal prey, i.e. spider mite eggs, was not influenced by the plant’s natural JA defences. We observed that the level of thrips-inflicted leaf damage, mite egg predation, thrips survival and -development, all did not differ when prey had been obtained from either WT or JA-impaired *def-1* tomato plants (Figs 3–6; diets with induced eggs versus with uninduced eggs, respectively). However, whereas thrips performance on a low-quality host plant increased significantly when *T*. *urticae* eggs were added, it decreased when *T*. *evansi* eggs were added, irrespective of the tomato genotype on which the eggs had been produced (Figs 4–6). Hence, although differences in the quality of eggs of the two-mite species had profound effects on thrips performance, these effects were independent from the plant’s JA defences and from whether or not their prey had induced or suppressed these.

Feeding by *F*. *occidentalis* has previously been shown to induce JA defences in Arabidopsis^59^, Chinese cabbage^50^, cotton^60^ and tomato plants^6,51^ and these responses have been associated with negative effects on the reproductive performance, population growth^50^ and feeding intensity of *F*. *occidentalis* adults. Artificially boosting JA defences was shown to affect the abundance^12^ and host-preference^51^ of adults but also the amount of feeding damage^10^ and development^52^ of larvae. However, unlike the adults the larvae of *F*. *occidentalis* were found to tolerate the naturally induced levels of JA-defences^6^ and this study. This indicates that artificially boosting JA-defences may sometimes overamplify rather than mimic the natural response. Notably, a difference in susceptibility of different arthropod life stages to JA defences was also reported for the omnivorous stink bug *Podisus maculiventris*, yet in the opposite direction^28^. No matter the cause, for our purpose, the fact that the larvae we used tolerate JA-regulated direct plant defences sufficed for testing if JA defences indirectly affected thrips behaviour and performance via changes in prey quality.

While thrips, including *F*. *occidentalis*, are known to often co-occur with- and prey on several species of spider mites^41,48,61^ to the best of our knowledge, though, this is the first report of *F*. *occidentalis* preying on eggs of *T*. *evansi* (Fig. 3). However, for *F*. *occidentalis* larvae, the *T*. *evansi* eggs were clearly not a good resource. Unlike with *T*. *urticae* eggs, thrips performance on sweet pepper leaves did not improve when *T*. *evansi* eggs were offered as additional food source. In fact, the percentage of larvae that reached adulthood within 15 days even significantly decreased (Fig. 4) and the same trend was visible for thrips survival (Fig. 5). This result is puzzling: *T*. *evansi* is an unsuitable prey for multiple carnivorous predators of *T*. *urticae*^62^, some of which nonetheless feed from *T*. *evansi* eggs in no-choice assays. In fact, predation of *T*. *evansi* eggs seems to be rare in nature^63,64^, suggesting that *T*. *evansi* may accumulate substances that make it toxic. There are indications that it may sequester toxic plant-derived metabolites, which are probably also passed on to their eggs, and that this ability confers protection against predation^62,64–66^. On the contrary, *T*. *urticae* and its eggs are highly suitable for a wide variety of predators^62,67^. Considering that these two spider mite species cope differently with the plant defence machinery, i.e. while *T*. *urticae* induces SA and JA plant defences *T*. *evansi* is able to suppress these, we hypothesize that its toxicity might have been evolved under pressure by the increased risk of predation that comes with defence suppression^8,68^. This implies that, if this toxicity can be attributed to sequestered plant substances, these most likely are constitutive defensive metabolites. The reduced consumption of *T*. *evansi* boosted eggs by thrips (Fig. 3b) may indicate that high amounts of JA increase the pool of these unknown metabolites. The fact that consumption of *T*. *urticae* eggs was not influenced by the JA-treatment strengthens the notion that this toxicity is an *T*. *evansi*-specific trait (Fig. 3a). Possibly, comparative metabolomics of *T*. *evansi* and *T*. *urticae* eggs from induced and boosted leaf material may reveal the causal agents of this phenomenon.

*F*. *occidentalis* is considered an opportunistic predator, meaning that it is an omnivore that does not actively search for mite prey and only feeds on it upon close encounter^48,69,70^. It is therefore, possible that its visual and/or olfactory sensory organs are insufficiently developed for accurately assessing the nutritional value of mite eggs, e.g. for discriminating eggs from *T*. *urticae* and *T*. *evansi*. At this moment, we can also not exclude the possibility that *T*. *evansi* eggs were pierced but not eaten and thus, that the decrease in thrips performance compared to the plant-only diet was caused by the time spent on handling eggs. Yet, we believe thrips larvae do eat these eggs, because boosted *T*. *evansi* eggs were preyed on less than the other eggs (Fig. 3b). However, since we did not observe a significant effect on thrips performance when feeding on boosted *T*. *evansi* eggs, the biological relevance of such behaviour remains unknown.

Considering the rapid induction of tomato JA defences by the spider mite *T*. *urticae*^53,71–73^ and the strong negative effects these defences have on adult performance^6,26,27^ and on egg hatching rate^74^, it is surprising that we did not observe any indirect effects of the defences on the performance and behaviour of thrips larvae when feeding on induced-, uninduced- or boosted *T*. *urticae* eggs. In earlier no-choice experiments with *Tetranychus pacificus* eggs as additional food source on non-induced cotton leaves, *F*. *occidentalis* larvae consumed less eggs produced on cotton previously infested (for three days) with *Tetranychus turkestani* compared to on non-infested cotton^75^. Cotton defences are known to be induced by *T*. *turkestani*^76^ and in the laboratory thrips caused less damage to cotton leaves after an earlier infestation with *T*. *turkestani*^29^. Accordingly, in field experiments, thrips avoided to colonize cotton plants previously infested with *T*. *turkestani*. However, they no longer did so when *T*. *pacificus* and its eggs were present on these induced plants^75^. This illustrates the complex interactions between the host plant, herbivorous spider mites and omnivorous thrips.

It is possible that JA-defence mediated indirect effects on mite eggs for thrips require more time to take effect and, hence, that the 24 h of mite infestation we used to produce prey eggs was insufficient to detect them. Although tomato plants may indeed activate and deactivate various defences throughout the course of a *T*. *urticae* infestation^53,73^, 24 h of infestation was previously sufficient to observe JA-defence related effects on the feeding behaviour of *Phytoseiulus longipes* preying on *T*. *urticae* eggs^8^. In addition, 24 h of infestation was also sufficient to observe changes in the feeding behaviour of *F*. *occidentalis* larvae preying on JA-boosted *T*. *evansi* eggs (Fig. 3b), further validating our experimental approach. Moreover, components of *T*. *urticae*’s diet can be incorporated into its eggs in as little as 6 h^77^. Together, this indicates that thrips larvae and predatory mites are differentially impacted by JA defence-mediated changes in *T*. *urticae* egg-quality suggesting that most of those effects will be direct (e.g. due to the transfer of tomato defence compounds to eggs) rather than indirect (e.g. due to changes in size or nutritional value).

Finally, we focussed on JA-defences in our study since it is clear that these defences strongly determine the ability of plants to cope with herbivores^1,78^. However, also other defences have distinct effects on tomato-mite interactions, such as salicylate-mediated defences^79^, acylsugars^80^, terpenes^81^, glycoalkaloids^82^ or methyl ketones^83^ which may cause changes in prey quality indirectly and, possibly independent from JA-defences, and may therefore not have been addressed by our experiments.

Taken together, feeding activities of thrips larvae – and possibly also adults – on spider mite prey seems to be determined predominantly by direct effects since naturally induced JA-defences did not affect the quality of the spider mite prey indirectly. Understanding the relative contribution of direct and indirect effects of plant defences on carnivores other than thrips, for example those used in biological control, may help to understand how top down control by natural enemies on plants carrying natural resistance comes about and how it can be facilitated.

## Methods

### Plants

Tomato (*Solanum lycopersicum* wild type, mutant *def-1* and transgenic *35 S::prosystemin*; all in the cv. Castlemart genetic background) and bean (*Phaseolus vulgaris* cv. Speedy) plants were germinated and grown in a greenhouse with 25/18 °C day/night temperatures, a 16:8 h (light:dark) photoperiod, and at 50–60% relative humidity (RH). Sweet pepper (*Capsicum annuum*) plants were germinated and grown in a climate room at 25 °C, 16:8 h (light:dark) and 60% RH (default settings). For experiments, we used 28 days old plants. Broadleaf cattail (*Typha latifolia*) pollen, manually collected in Amsterdam (The Netherlands), were dried in a stove at 40 °C for three days, sieved and stored at 4 °C until they were used in the experiments. All experiments were carried out in a climate room, to which tomato plants were transferred two days in advance.

### Spider mites

The *Tetranychus urticae* Santpoort-2 and *Tetranychus evansi* Viçosa-1 strains used for this study were reared in a climate room on detached leaves of bean and tomato, respectively. This *T*. *urticae* strain has been described before as an inducer of tomato JA defences, to which it is also susceptible, whereas the *T*. *evansi* strain has been demonstrated to suppress these defences^53^. For the experiments, adult female mites were randomly taken from the respective rearings and *T*. *urticae* females were habituated on tomato plants for three days to exclude possible effects of the bean diet on the composition of their eggs^77^.

### Thrips

*Frankliniella occidentalis* was obtained from Koppert Biological Systems (Berkel en Rodenrijs, the Netherlands) and it is reared on bean pods and broadleaf cattail pollen in a climate room ever since. For experiments, we used thrips larvae of a similar age (first instar, L1), these were obtained via generation of an “egg-wave” on bean pods only, i.e. adult female thrips were allowed to produce eggs for 24 h and the offspring was used for experiments four days later. In pilot experiments we had observed that larvae of this thrips strain performed equally well on wild type (WT) and *def-1* tomato plants, similar to the observations of Li *et al*.^6^. We verified these findings in additional bioassays (see below).

### Spider mite egg production on detached tomato def-1 leaflets treated with JA and Ile

The *def-1* mutant is highly susceptible to arthropod herbivores, including spider mites, as it is impaired in mounting JA defences upon herbivory, but exogenous application of JA can rescue this phenotype^6,55^. Here we restored JA defences in *def-1* by supplementing detached leaflets with JA and Ile as previously described and validated by Ataide *et al*.^8^. In short, *def-1* leaflets were placed with their petiolule in a 15 mL conical centrifuge tube (Greiner Bio-One, Kremsmünster, Austria) containing 0.05 mM (±)-JA and 1 mM Ile (Sigma-Aldrich, St. Louis, MO, USA) in tap water. Wild type and *def-1* leaflets in tubes with tap water served as controls. Tubes containing 1 mM Ile in tap water were not included, because this treatment had previously been shown not to affect JA defences^8^. Detached *def-1* leaflets were incubated in the JA + Ile solution for 24 h (henceforth referred to as ‘JA-treated *def-1*′), after which 15–30 adult female (tomato-habituated) spider mites were introduced to each leaflet. The number of mites was not exactly controlled because the goal was simply to collect eggs. A thin layer of insect glue (Bio-controle, São Paulo, Brazil) mixed with lanolin (Sigma-Aldrich) (v/v; 50/50) was deposited around the petiolule to prevent mites from escaping. Mites were allowed to produce eggs for 24 h (leaflets were still in the JA + Ile solution), after which these eggs were used for thrips bioassays (see below).

### Thrips bioassays

Two kinds of thrips bioassays were performed. In the first set of experiments we substantiated our initial observations that thrips larvae performed equally well on WT and *def-1* tomato plants by performing a feeding assay on leaf tissue from WT, *def-1*, JA-treated *def-1*, and transgenic *35S::prosystemin* (PS) plants. Leaf tissue consumption by *F*. *occidentalis* correlates well with reproduction and number of individuals^50^ and has been widely used as a proxy for determining the level of resistance of this omnivore to plants and their defences. After 24 h of incubating detached tomato leaflets in tap water (WT, *def-1*, PS) or tap water + JA + Ile (*def-1*), as described above, two or three leaf discs (d = 9 mm) were prepared from each leaflet, depending on their size. Leaf discs (adaxial side up) were placed on water-saturated cotton wool and infested with a single thrips larva. Three days later, the larva was removed and the leaf disc was scanned using a HP ScanJet 3570c Scanner (Hewlett-Packard, Palo Alto, CA, USA) for the *in silico* quantification of feeding damage^72^. These experiments were carried out in three blocks (experimental replicates) in time with in total n = 30 per treatment, except for experiments with PS leaflets, which were carried out in two blocks in time with in total n = 10.

In the second set of experiments we assessed thrips feeding behaviour and performance on sweet pepper leaves supplemented with eggs from spider mites that had been feeding from WT tomato (henceforth referred to as ‘induced eggs’), *def-1* (‘uninduced eggs’) and JA-treated *def-1* (‘boosted eggs’). Using a fine paintbrush, induced-, uninduced- or boosted eggs from either *T*. *urticae* or *T*. *evansi* were gently transferred to sweet pepper leaf discs (d = 9 mm). Sweet pepper is a low quality host plant for thrips, which therefore maximizes its consumption of alternative food, if available^47^. Leaf discs (adaxial side up) were placed on water-saturated cotton wool. A surplus of spider mite eggs was transferred to each leaf discs: this means either 30 *T*. *urticae* eggs or 20 *T*. *evansi* eggs (these numbers were chosen based on preliminary experiments and the availability of intact prey eggs was verified after each experiment). Initial attempts to offer eggs without plant material failed because of high larval mortality rates. Sweet pepper leaf discs without spider mite eggs, and sweet pepper leaf discs supplemented with broadleaf cattail pollen, i.e. a high quality food source for thrips^57^, were used as controls. A single thrips larva was transferred to each leaf disc. Subsequently, larval food intake (leaf area damaged, number of mite eggs eaten), development and survival were monitored for 15 days, i.e. until most surviving thrips had reached adulthood. Each larva was transferred to a new, but identically treated, leaf disc every three days. Immediately after larval removal, leaf discs were scanned to assess the amount of feeding damage, as described earlier. Larval survival and developmental stage were scored daily. For logistical reasons, experiments to assess thrips behaviour and performance with eggs of *T*. *urticae* as additional food source versus with eggs of *T*. *evansi* as additional food source (and their respective controls) were performed at different moments in time. Experiments using *T*. *urticae* eggs were carried out in three blocks (experimental replicates) in time with in total n = 30 per treatment, experiments using *T*. *evansi* eggs were carried out in four blocks in time with in total n = 45 per treatment.

### Statistical analysis

All statistical analyses were performed with the software R, version 3.3.3^84^. Total amount of feeding damage (mm^2^) and total number of spider mite eggs consumed per surviving thrips were analysed using linear mixed-effects models (LMER) in the lme4 package^85^. Thrips developmental time (days), thrips survival, and proportion of thrips reaching adulthood, were analysed using cox mixed-effects models (CoxME) in the coxme package^86^. All the fixed factors were individually included in the model as the response variable (*y*), and treatment was included as the explanatory variable (*x*). Experimental replicate was included as a random factor in the model. Prior to analysis, data was inspected for homogeneity of variances and normality of residuals and, when necessary, data was log-transformed to fit it into a normal error distribution. When significant differences among treatments were found, contrast analyses were performed by amalgamating levels as long as this did not produce a significant (*P* ≤ 0.05) change in deviance^87^.

## Data Availability

The datasets generated and analysed during the current study are available in the figshare repository, https://figshare.com/s/4edf9fed276ca8e7fcec.
